# The Impact of the Covid‐19 Pandemic on Current Anatomy Education and Future Careers: A Student’s Perspective

**DOI:** 10.1002/ase.1966

**Published:** 2020-05-05

**Authors:** Thomas Franchi

**Affiliations:** ^1^ Medical Student The Medical School The University of Sheffield Sheffield United Kingdom; ^2^ Human Anatomy with Education Student Department of Biomedical Science The University of Sheffield Sheffield United Kingdom

To the Editor, *Anatomical Sciences Education:*

As both an anatomy student and anatomy demonstrator, *Anatomical Sciences Education* has become my go‐to journal to further my knowledge of the latest developments in anatomy education, innovations in pedagogical practice, and indeed perspectives on current affairs in the field. The world is experiencing an ongoing and serious pandemic, and I felt it necessary to comment, from the perspective of a student, on the impact this pandemic has had and is having on students’ anatomical education, and my concerns about the implications it might have on the future of current anatomy students. In the remainder of this letter, I wish to present some of my reflections on this matter.

Readers will be aware that the outbreak of the novel coronavirus began in Wuhan, China, in late December 2019 (Zhu et al., 2020), and spread exponentially in our age where increasing urbanization and frequent international travel allow for the uninterrupted transmission of infectious diseases (Alirol et al., 2011). The first reported case in the United States was on 20th January 2020 (Holshue et al., 2020), and the first cases in the United Kingdom was detected on 31st January (Moss et al., 2020). The World Health Organization named the disease caused by this severe acute respiratory syndrome coronavirus 2 (SARS‐CoV‐2) as Covid‐19 on 11th February (WHO, 2020a), and subsequently labeled it a pandemic on 11th March (WHO, 2020b). On 2nd April, the number of cases reported worldwide crossed 1 million, with 205 countries and territories affected (Worldometer, 2020).

As governments struggle to contain the vicious spread of Covid‐19, and with over a third of the world’s population currently under some form of lockdown (Kaplan et al., 2020), the effects the virus has had on people’s daily lives is clearly like nothing most people have experienced before. One of the many affected sectors is education (UNESCO, 2020). In the United Kingdom, the majority of universities and medical schools had suspended face‐to‐face teaching by 17th March 2020 (Staton and Jack, 2020), forcing students to move to online distance learning for the indefinite future.

Pandemics are not a new occurrence. Indeed, Galen of Pergamon documented a 20‐year smallpox pandemic starting in 166 AD (Mattern, 2011), and more recently the world experienced the 2003 severe acute respiratory syndrome (LeDuc and Barry, 2004) and 2009 H1N1 swine flu pandemics (Collignon, 2011). Although the Covid‐19 pandemic is likely to be the first which current anatomy students are affected by, lessons and experiences can be drawn from the previous crises to help us adapt and continue education. For example, web‐based learning was already successfully used in the 2003 pandemic to minimize the reduction in education that students received (Patil et al., 2003; Lim et al., 2009), and is once again being used to great effect (Lim, 2020). It is of course entirely possible for students to learn anatomy without a cadaver and solely from textbooks and online resources (McMenamin et al., 2018), and indeed a number of medical schools no longer use cadaver dissection (Patel et al., 2015). However, the disappeared practical teaching at the hands of Covid‐19, regardless of whether students normally receive cadaveric teaching or not, will most likely have many lasting impacts on students. In light of the learning environment now being far less than optimal, the loss of face‐to‐face contact and direct interactions with both peers and teachers may potentially stunt students’ development as anatomists. Despite the advances in technology that allow for online distance learning, acquiring anatomical knowledge in the laboratory, ideally through cadaver dissection, is often still regarded as not only a rite of passage but also the most effective method (Ghosh, 2017).

When students lost access to dissection rooms, they lost access not only to cadavers, but also to a range of other optimal learning modalities: prosections, models, pathology specimens, skeletons, and others (Sugand et al., 2010). Previous authors have highlighted that the modern medical curriculum already restricts students’ exposure to anatomy (Warner and Rizzolo, 2006), and indeed this pandemic has further shortened the contact time current students have received. As a result, current anatomy students are being taught anatomy without access to practical‐based learning materials, be that cadavers, prosections, or models. Anatomy learning without cadavers is a practice which is generally seen as less favorable, but one which has arguable merits and has been used as standard in many institutions (McLachlan et al., 2004), but when prosections, models, and other learning materials are also removed, learning becomes difficult. Adaptation to online distance learning is no easy task for students or teachers, and simply providing an online atlas is unlikely to provide students with an “appreciation for the fabric of the human body” (Gregory and Cole, 2002). Despite there being a large number of online anatomy software programs available for students to use, they can often be costly. Institutions that can afford to do so should endeavor to give their students access to these during the current situation. However, to account for equality of opportunity between institutions with varying financial freedom, I would implore software companies to consider providing all anatomy students with temporary free access to their programs during the pandemic. Having said this, previous studies have demonstrated that, despite being useful, online programs provide significantly lower rates of self‐perceived learning and satisfaction compared to dissection (Mathiowetz et al., 2016). There is also a steep learning curve associated with using these programs for both teachers and students (Doubleday et al., 2011), with many students finding it difficult to manipulate models and focus on structures of interest (Attardi et al., 2016), thus further bringing into question their usefulness in times as challenging as a pandemic. However, studies investigating the efficacy of purposely designed, solely online programs have not yet been done. Indeed, the Covid‐19 crisis may serve to inform us on whether such approaches are able to deliver appropriate learning gain. Further, if these online programs were suitably integrated into curriculum design and used to carefully guide students through a learning journey, rather than simply made available as another resource, then perhaps these tools could prove very beneficial.

Mixed methods of teaching and learning anatomy in the current crisis are clearly needed. The addition of instructional dissection videos goes part of the way toward normality, where students can essentially watch a prerecorded dissection taking place (Langfield et al., 2018). Indeed, even direction toward appropriate YouTube videos can help students to understand anatomical concepts (Jaffar, 2012). In conjunction with online digital photographs of cadavers, interactive anatomy images, and the provision of self‐testing tools (O’Byrne et al., 2008), students may start to feel supported in their online distance learning. The importance of personal online interactions cannot be overlooked however, and attempting to reduce the distance between learners through provision of chat rooms or real‐time tutorials is a key element to successful online learning (Stone and Barry, 2019). It would seem that a purposely designed online course which integrates a number of elements into a learning journey would potentially provide a solution to the current pause in face‐to‐face teaching. Further, a modern ideal for home learning of anatomy would be in the form of virtual reality (VR) resources (Erolin et al., 2019), and although we are not quite yet in the age where this technology is a household staple, there are elements of it which could be adapted to a remote learning environment. Indeed, most smartphones are compatible with Google Cardboard allowing students to experience VR from their own phones, if provided with suitable software (Izard et al., 2017).

The implication the pandemic has on summative assessment is a further worry for students. Among the plethora of modalities used to assess anatomy are the written spotter examination (Smith and McManus, 2015) and oral *viva* (Evans et al., 2014). However, these methods can obviously not be used in the current situation. With a move to online examinations seemingly inevitable for this year’s global cohort of anatomy students, I question their preparedness for this form of assessment and wonder whether students will perform to the standard that they might have if their examinations were in the modality they were planned to be. This being said, studies have demonstrated that students tend to score similarly regardless of whether the examination is practical or online (Inuwa et al., 2012). Although many students already use anatomy flash cards and digitized spotter‐like tests in their learning, I would call on institutions to provide students with clear guidance on the adjusted format of their examinations and to provide ample opportunity for no‐stakes practice of these new modalities. Indeed, it has been raised by previous authors that traditional spotter examinations are arguably not an effective assessment technique, as they focus almost entirely on testing a students’ ability to recall information (Choudhury et al., 2016). Therefore, perhaps the Covid‐19 pandemic presents institutions with an opportunity for innovation in assessment approaches that allow for accurate representation of both a student’s knowledge and understanding of anatomical sciences.

Aside from the challenges relating to continuing students’ anatomical education online, the Covid‐19 pandemic also raises issues relating to current anatomy students’ futures. Students consider working with cadaveric material a crucial part of their development toward becoming a professional in the field (Smith et al., 2014), be that as a doctor, dentist, or biomedical scientist. Clinically meaningful learning of anatomy is crucial to students’ understanding of the relevance of their knowledge to future practice (Collins, 2008). As such, students’ clinical understanding and appreciation for the relevance of anatomy might well suffer due to the current lack in practical teaching, at the detriment to their future, and so adaptive institutions must ensure that online learning resources do not lose this important clinical relevance (Turney, 2007). As an aspiring surgeon, I am particularly concerned about the reduction in dissection experience that current anatomy students received (Drake et al., 2014). Cadaver dissection is an invaluable opportunity for the development of fine motor skills in a stress‐free environment (Krähenbühl et al., 2017), and so I wonder what implications this might have on the future of students in similar positions to myself. Indeed, this worry extends further to the future of surgery in a much wider sense. Poor anatomy teaching at medical school is often cited by students as a reason for not considering a surgical career (Cooper and Gray, 2014), and although the current situation of anatomy teaching is not intentional, it is possible that the quality of teaching that students are now able to receive may be of lower than prior to the pandemic. Whether the pandemic causes a drop in applications to surgical training posts for this year’s students will not be known for many years, but abandoning dissection has proven detrimental to the competency of future surgeons (Memon, 2018). A further compounding factor on this issue is the fact that many students discover their love or natural talent for surgery as an anatomy student—“Gross anatomy […] may also be an unrecognized fork in the road in [students’] pursuit of choosing a medical specialty” (Archibald and Carlson, 2009). Perhaps through this disappeared practical teaching, we are losing the opportunity to discover the next top surgeons of our time, or creating students that had a distinct gap in their anatomical understanding.

For those students who wish to become future anatomy teachers, the lack of complete and ongoing exposure to a variety of teaching and learning techniques may well impact on the styles and methods they will later employ as teachers. Without giving them the opportunity to consider best teaching practices from their perspectives as students (Estai and Bunt, 2016), I question whether they will feel suitably prepared to enter the employment market with the confidence that they possess not only the anatomical understanding but also the pedagogical experience to become effective educators of the future. On the other hand, students in this situation might wish to seize the opportunity to expose themselves to a broader range of teaching techniques than they might have otherwise encountered. Many institutions worldwide have now made prerecorded lectures freely available to the general public, and so students could experience other teachers’ styles from around the world. Alternatively, students could experiment with online peer teaching groups, and take turns delivering a short anatomy tutorial to their peers through a video conference with the opportunity to receive feedback on the effectiveness of their teaching style. For those more inclined toward practical work, students could experiment with more artistic techniques, such as body painting, anatomical drawing, clay work, or the use of pipe cleaners in order to see what techniques work for them and which they therefore may wish to trial when teaching (McMenamin, 2008; Lefroy et al., 2011; Kooloos et al., 2014).

There are also a number of practical issues to consider as a result of the pandemic. With students no longer allowed to attend face‐to‐face teaching, the cadavers they were working on may now not be fully utilized, depending on individual institutional set‐up. Aside from the many implications this has on students’ learning, as outlined above, it is also a sad situation as it is not what the donors wanted. However, perhaps dissection laboratory staff could utilize these cadavers for making prosection materials, creating image libraries, or using them for specialist short courses, in order to ensure that the donors are used for the highest education benefit which the circumstances allow. An argument could of course be made for allowing students to complete their dissection in the new academic year, and indeed most embalming techniques would allow for the cadavers to still be usable by that time (Brenner, 2014), however this is clearly logistically difficult from both storage space and time commitment perspectives, and so would vary in viability between institutions. Indeed, even if this provision were possible, graduating students would still miss out. It is important to note that missed practical experiences are a disappointment to both students and their teachers, not just the students. What is clear from the contributions regularly published in *Anatomical Sciences Education* is that the anatomical community is a very creative and adaptive one. Anatomy educators will undoubtedly do their upmost to accommodate students who wish to regain at least part of the cadaveric experience which Covid‐19 has caused them to lose, and this is something which students can take comfort in.

The immediate future of dissection is called into question also, with the indefinite suspension of the vast majority of body donation schemes to universities and hospitals (HTA, 2020). It is obvious that this will result in a severe shortage of donor bodies for the incoming academic cohorts, which in turn will have significant influence on the modality and quality of teaching which they will receive. With the potential risks associated from coming into contact with people who died from Covid‐19 (Finegan et al., 2020), it is unclear when and how body donation schemes will restart. Nevertheless, even when the Human Tissue Authority was introduced in the United Kingdom in 2004 following a national scandal of organ retention without consent (Sheach Leith, 2007), the drop in number of body donors still recovered, so we can only assume that they will do so again after this crisis. This issue does however lead me to question whether this pandemic may leave us with lasting change on how anatomy education and indeed wider university education is carried out (Jones, 2020), just as it is likely to leave us with a realization that many of our social norms are obsolete, like traveling to work at an office (Hern, 2020).

Potential educational disruption and uncertainty about students’ futures are no doubt two of the unavoidable by‐products of the pandemic we currently live through, but there is also a more fundamental emotional experience which many anatomy students may now be facing. It is not just anatomy which students learn from the body donors. Indeed, students develop personal and professional competencies through interactions with their donor (Weeks et al., 1995), and build a certain special emotional relationship with them over the course of their program. The fact that students learn a whole range of nontraditional discipline‐independent skills (NTDIS) through their study of and interaction with anatomy should not be disregarded (Evans and Pawlina, 2015). For students who learn on cadavers, the donor is their first patient, and for those who learn anatomy through modalities, the use of human representations symbolizes the future patient (Evans et al., 2018). Students’ internal and external development of emotional intelligence, situational awareness, and professional behaviors, as well as personal feelings of love and empathy are all catalyzed in the anatomy laboratory through interactions with peers, teachers, technicians, academics, and the donors. It is clear that anatomy curricula teach students much more than just anatomy, and NTDIS are a crucial element of this (Evans and Pawlina, 2015). In these challenging times, it is therefore especially important for students to be aware of their NTDIS sets, and to be adaptable and resilient to their circumstance (Evans et al., 2018).

As a result of prematurely leaving the laboratory, I fear many students will be left feeling guilty that they have not yet had the opportunity to say thank you and goodbye to their donor bodies—“a necessary ritual for students” (Boeckers and Boeckers, 2016). Although there will be opportunities for students to pay their respects, these will undoubtedly not occur in the usual manner and so may not serve their full purpose for some students. I therefore wish to end this letter by asking students to spend some time reflecting on their experiences in the anatomy laboratory prior to the Covid‐19 pandemic, and to look forward to the time when they will be allowed to return to it.
